# Noninvasive approach to indicate risk factors of nonalcoholic steatohepatitis overlapping autoimmune hepatitis based on peripheral lymphocyte pattern

**DOI:** 10.1007/s00535-023-02038-y

**Published:** 2023-09-14

**Authors:** Akira Kado, Takeya Tsutsumi, Hiroshi Yotsuyanagi, Kazuhiko Ikeuchi, Kazuya Okushin, Kyoji Moriya, Kazuhiko Koike, Mitsuhiro Fujishiro

**Affiliations:** 1 Department of Gastroenterology, Graduate School of Medicine, The University of Tokyo, 7-3-1 Hongo, Bunkyo-ku, Tokyo, 113-8655 Japan; 2 Division for Health Service Promotion, The University of Tokyo, 7-3-1 Hongo, Bunkyo-ku, Tokyo, 113-8655 Japan; 3 Department of Infection Control and Prevention, Graduate School of Medicine, The University of Tokyo, Tokyo, Japan; 4 Division of Infectious Diseases, Advanced Clinical Research Center, Institute of Medical Science, The University of Tokyo, 4-6-1 Shirokanedai, Minato-ku, Tokyo, 108-8639 Japan; 5 Department of Infectious Diseases, Graduate School of Medicine, The University of Tokyo, 7-3-1 Hongo, Bunkyo-ku, Tokyo, 113-8655 Japan; 6 Department of Gastroenterology, Kanto Central Hospital, 6-25-1 Kamiyoga, Setagaya-ku, Tokyo, 158-8531 Japan

**Keywords:** Flow cytometry, Nonalcoholic steatohepatitis, Autoimmune hepatitis, Peripheral lymphocyte frequency, Peripheral blood mononuclear cells

## Abstract

**Background:**

Nonalcoholic fatty liver disease (NAFLD)/nonalcoholic steatohepatitis (NASH) clinically includes autoimmunity as indicated by antinuclear antibody (ANA) positivity and overlap of autoimmune hepatitis (AIH). Discriminating AIH-overlap NASH from NAFLD/NASH is required for proper treatment, and typically involves pathological diagnosis by invasive liver biopsy. Differential patterns of peripheral lymphocytes in NAFLD and AIH were assessed to noninvasively indicate risk factors of AIH-overlap NASH by flow cytometry (FCM).

**Methods:**

We assessed the differential frequencies of peripheral lymphocytes in 115 patients: 70 NASH (ANA negative:positive:AIH-overlap = 36:20:14), 18 NAFL, and 27 AIH (acute:chronic = 12:15) patients diagnosed by FCM. We focused on the following populations of lymphocytes: T cells, B cells, natural killer (NK) cells, NKT cells, helper T cell (Th) subsets (Th1, Th2, and Th17), and regulatory T cells; we also examined programmed cell death (PD) 1 and cytotoxic T-lymphocyte antigen levels.

**Results:**

Several significant differences in laboratory parameters and peripheral lymphocyte frequencies were found among the NAFLD and AIH subgroups. In univariate and multivariate analyses, hyaluronic acid level, liver stiffness, and the frequencies of Th17 and CD8^+^ PD1^+^ T cells were independent risk factors of NASH in NAFLD. Regarding overlap of AIH, only the frequency of CD8^+^ PD1^+^ T cells (odds ratio, 0.01; 95% CI 0.00–38.9, *p* = 0.004) was an independent risk factor in NASH and significantly decreased in AIH.

**Conclusions:**

The decreased frequency of peripheral CD8^+^ PD1^+^ T cells is an independent risk factor of NASH overlapping with AIH in the present cohort. Our findings will facilitate development of a new noninvasive FCM method for indicating risk factors of NASH, including autoimmunity.

**Supplementary Information:**

The online version contains supplementary material available at 10.1007/s00535-023-02038-y.

## Introduction

Nonalcoholic fatty liver disease (NAFLD) leads to various metabolic disorders, such as diabetes mellitus and dyslipidemia [1]. In NAFLD, nonalcoholic steatohepatitis (NASH) is progressive and leads to liver cirrhosis (LC) and hepatocellular carcinoma (HCC) [2]; early diagnosis and treatment of NASH are required to prevent its progression to fatal conditions [3]. Furthermore, antinuclear antibody (ANA), which is associated with autoimmune diseases, is positive in 20–30% of patients with NASH, and is reportedly related to its progression and autoimmune mechanism [4]. The pathologies of NASH include autoimmunity and infrequently overlap with autoimmune hepatitis (AIH) [5]. In AIH-overlap NASH, depending on pathological diagnosis by liver biopsy, early steroid therapy is required; however, this can worsen the pathology of NASH [6]. The selection of NASH cases suspected of overlapping with AIH prior to pathological diagnosis is vital; especially, ANA-positive NASH can be misdiagnosed as AIH-overlap NASH, and invasive percutaneous liver biopsy is time-consuming [7, 8]. Reportedly, ANA is occasionally seen in liver diseases other than NAFLD and represents B cell activation; the presence of ANA does not necessarily suggest overlapping AIH [9–11]. The clinical background of AIH-overlap NASH should be elucidated, and a noninvasive approach to indicate risk factors of NASH overlapping with AIH prior to liver biopsy is needed.

Various noninvasive diagnostic procedures or biomarkers for NASH, depending on pathological diagnosis, have been reported [12–15]. We reported a noninvasive diagnostic method for NASH based on gene expression levels in peripheral blood mononuclear cells (PBMCs); peripheral lymphocyte patterns had the potential for predicting NASH [12]. Several peripheral lymphocyte pattern predictors of hepatic and autoimmune diseases have been examined [16]. NASH has been suggested to involve peripheral immune activation, suppression, and tolerance by T cells, B cells, natural killer (NK) cells, and NKT cells [12, 17, 18]; the same is true for AIH [19]. NASH and AIH have differential hepatic pathologies; a few flow cytometry (FCM) studies have focused on differential patterns of peripheral lymphocytes in NASH and AIH [19–21]. However, few studies have evaluated these patterns as predictors of NASH and AIH [22]. Furthermore, no studies have assessed differential patterns of peripheral lymphocytes in NASH overlapping with AIH.

Clinically, NAFLD is encountered more frequently than NASH; therefore, NASH cases suspected of overlapping with AIH should be selected from among NAFLD/NASH cases. In this study, we evaluated the differential patterns of peripheral lymphocytes between NASH and non-NASH and assessed these differential patterns as noninvasive indicators of NASH overlapping with AIH.

## Methods

### Patients and PBMC samples

From 2016 to 2019, percutaneous liver biopsy was performed on patients with clinically suspected NASH and/or AIH at the University of Tokyo Hospital. Among them, 115 patients provided written informed consent, and PBMCs were obtained from 88 patients (NASH:nonalcoholic fatty liver [NAFL] = 70:18) pathologically diagnosed as NAFLD, and from 27 patients diagnosed with AIH (acute:chronic = 12:15), not overlapping with NASH (Supplementary Fig. 1). The 70 NASH patients were divided into three groups: ANA negative, 36; ANA positive, 20; and AIH-overlap, 14. The patients met the following criteria: the levels of serum aspartate aminotransferase (AST) or alanine aminotransferase (ALT) above the upper limit of normal for ≥ 6 months; alcohol consumption < 30 g/day for men and < 20 g/day for women [8]; seronegativity for hepatitis B virus surface antigen and hepatitis C virus antibody and the absence of other liver diseases, such as primary biliary cholangitis (PBC), primary sclerosing cholangitis, and drug-induced liver injury (DILI); and no history of autoimmune disease except AIH, chronic inflammatory disease except hepatitis, and malignant tumor, because these comorbidities may affect peripheral lymphocyte patterns. Patients with NAFLD also met the criterion of enhanced hepatorenal contrast by abdominal ultrasonography (AUS). Although there were no specified guidelines or consensus on the diagnosis of AIH-overlap NASH [4, 7], in this study, patients suspected of overlapping AIH met at least one of the following criteria: serum ALT level 1.5 times higher than 6 months ago; ANA > 1:40; serum immunoglobulin (Ig) G level > upper limit of normal within 6 months [23]. Patients suspected of acute or chronic AIH alone also met at least one of the above criteria, and acute AIH was defined by the presence of acute-onset symptoms (e.g., jaundice, fatigue, right upper quadrant pain, and/or anorexia) for less than 6 months in combination with a total bilirubin level of > 5 mg/dL and/or a serum ALT level of greater than 10 times higher than the upper limit of normal [24]. The exclusion criteria were symptoms of viral or bacterial infection, malignant tumor or chronic inflammatory disease, and immunosuppressant use. Eighteen subjects who did not undergo liver biopsy were included as controls in the non-NASH group and met all of the following criteria: normal values of serum AST and ALT; no history of autoimmune disease, chronic inflammatory disease, or malignant tumor; and absence of NAFLD according to AUS. In total, 133 PBMC samples were separated from blood using a Ficoll Kit [12]. Regarding AIH-overlap NASH, PBMCs were obtained from 9 of 14 patients again after 1 year; these 9 patients, who could be followed up on steroid treatment in this study, underwent biochemical remission, compared before and after the treatment. Biochemical remission was defined as the complete normalization of aminotransferases and/or IgG [25]; all these 9 patients were free of steroid treatment after 1 year. This study was approved by the Ethics Committees of The University of Tokyo and was performed in accordance with the Declarations of Helsinki.

### Clinical metabolic and autoimmune parameters

Comorbid diseases (immunological conditions in particular) and drug intake were recorded, and body mass index (BMI) was examined on the day of liver biopsy. Blood samples were obtained for biochemical tests for albumin, AST, ALT, γ-glutamyl transpeptidase (GGT), total bilirubin, high-density lipoprotein (HDL-C), low-density lipoprotein (LDL-C), triglycerides, creatinine, C-reactive protein (CRP), number of platelets, prothrombin time, fasting blood glucose (FBG), hemoglobin A1c (HbA1c), ANA, anti-mitochondrial antibody (AMA) -M2, IgA, IgG, IgM, and hyaluronic acid. ANA, AMA-M2, IgA, IgG, and IgM are serological hallmarks for diagnosis of autoimmune liver disease (AILD), such as AIH and PBC [26, 27]. Liver stiffness and controlled attenuation parameter (CAP) were also measured to assess fibrosis and steatosis, respectively, with transient liver elastography performed by FibroScan (FS) [14]. In control subjects clinically not suspected to have NAFLD and AIH, the ANA, AMA, IgA, IgG, IgM, hyaluronic acid, and liver stiffness levels and CAP values were not determined.

### Histological assessment

Liver biopsies were examined by trained, experienced hepatopathologists blinded to the clinical data and study design. Significant hepatic steatosis was considered present when ≥ 5% of hepatocytes exhibited fatty changes, and NASH liver histology was assessed according to the Matteoni classification [26, 28]. NASH progression was assessed by determining the NAFLD activity score (NAS) based on the degrees of steatosis, lobular inflammation (LI), and hepatocellular ballooning [29]. Pathological differences in NAS and fibrosis were assessed in patients with NAFLD. As diagnostic criteria of AIH, the revised International Autoimmune Hepatitis Group (IAIHG) scoring system was adopted [30], and especially, regarding liver histology, the cases with all pathological findings of interface hepatitis, lymphocytic infiltration, plasma cell infiltration, rosette formation, and emperipolesis were adopted in this study [31, 32]. Only 41 patients diagnosed with definite AIH (total IAIHG score ≥ 15) were enrolled, and all underwent pathological diagnosis by liver biopsy. Among these 41 patients, 14 with histological features of both NASH and AIH were defined as AIH-overlap NASH, although there were no established diagnostic criteria for AIH-overlap NASH [4, 7, 23]. NASH patients without distinct histological features of AIH were excluded from diagnosis of AIH-overlap, because severe portal inflammatory infiltrate in AIH-overlap NASH may obscure these features [33]. The NAFL patients had no distinct histological features of AIH, and patients not diagnosed with NAFLD and/or AIH were excluded.

### Surface staining and flow cytometric analysis

**Fig. 1:**
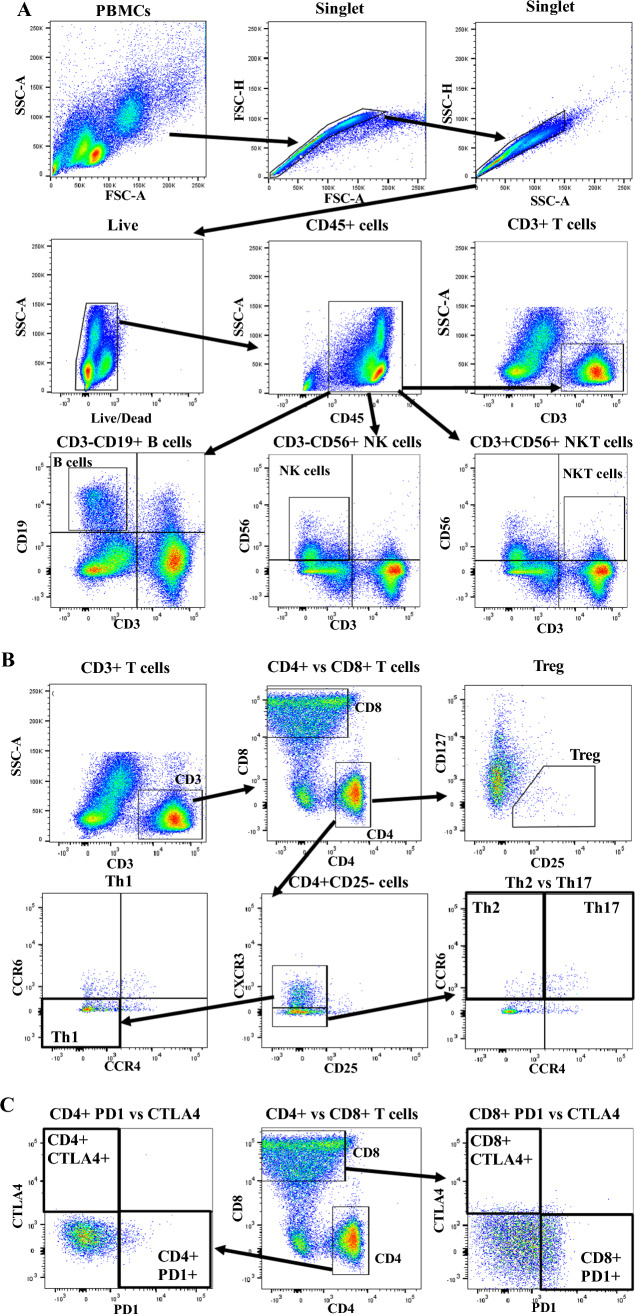
FCM plots and gating strategies used to identify lymphocytes: single and viable cells, followed by the selection of CD45 + leukocytes; next, followed by the selection of (**A**) CD3^+^ T cells, CD3^−^ CD19^+^ B cells, CD3^−^ CD56^+^ NK cells, and CD3^+^ CD56^+^ NKT cells; (**B**) CD4^+^ T cells, CD8^+^ T cells, CD25^−^ CXCR3^+^ CCR4^−^ CCR6^−^ Th1, CD25^−^ CXCR3^−^ CCR4^−^ CCR6^+^ Th2 cells, CD25^−^ CXCR3^−^ CCR4^+^ CCR6^+^ Th17 cells, and CD25^+^ CD127^−^ Tregs; (**C**) CD4^+^ PD1^+^, CD4^+^ CTLA4^+^, CD8^+^ PD1^+^, and CD8^+^ CTLA4^+^ T cells. Data are from one healthy control

PBMCs were processed as shown in the Supplementary Methods for surface staining of human peripheral lymphocytes based on two 11-color antibody cocktails (Supplementary Table 1). Subsequently, they were processed for FCM using fluorescence-activated cell sorting (FACS) Aria (BD Biosciences, San Jose, CA). FCM data were exported and analyzed using FlowJo version 10.8.1 (Treestar). FCM plots and representative gating strategies were used to identify lymphocytes as shown in the Supplementary Methods (Fig. 1). CD45-based gating was used to assess lymphocyte frequencies, because CD45 is a surface marker for total leukocytes [34]. CD45^+^ leukocytes were selected, followed by CD3^+^ (whole T cells), CD4^+^ CD8^−^ (helper T cells, Th), CD4^−^ CD8^+^ (cytotoxic T cells), CD3^−^ CD19^+^ (B cells), CD3^−^ CD56^+^ (NK cells), and CD3^+^ CD56^+^ (NKT cells) as shown in Fig. 1A and using Cocktail I, as summarized in Supplementary Table 1 [34–36].

Th subsets were examined as contributors to the pathologies of autoimmune and inflammatory diseases [37]. Among Th subsets, CD25^−^ C–X–C chemokine receptor (CXCR) 3^+^ C–C chemokine receptor (CCR) 4^−^ CCR6^−^ Th1, CD25^−^ CXCR3^−^ CCR4^−^ CCR6^+^ Th2, CD25^−^ CXCR3^−^ CCR4^+^ CCR6^+^ Th17, and CD25^+^ CD127^−^ regulatory T cells (Tregs) were selected using the gating strategies in Fig. 1B and Cocktail II (Supplementary Table 1) [36, 38]. Tregs maintain immune homeostasis and prevent autoimmune diseases; i.e., they regulate immune tolerance [39]. Programmed cell death (PD) 1 and cytotoxic T-lymphocyte antigen (CTLA) 4 surface markers on CD4^+^ and CD8^+^ T cells have been implicated in oncology and autoimmune diseases [40, 41]. Indeed, PD1 expression can cause life-threatening autoimmune diseases by disrupting T-cell homeostasis [19, 20, 40]. CD4^+^ PD1^+^, CD4^+^ CTLA4^+^, CD8^+^ PD1^+^, and CD8^+^ CTLA4^+^ T cells were selected using the gating strategies described in Fig. 1C and Cocktail II (Supplementary Table 1).

CD45 can be used to calculate the frequencies of T cells, B cells, NK cells, and NKT cells; CD4 can be used to calculate those of Th subsets. Based on PD1 and CTLA4 expression, T lymphocyte frequencies were calculated by CD4- and CD8-based gating. Target lymphocytes in 133 PBMC samples were selected using the gating strategies shown in Fig. 1, and lymphocyte frequencies were calculated.

### Statistical analysis

Statistical analysis is described in the Supplementary Methods.

## Results

### Patient characteristics

By liver biopsy, 88 of 115 patients were diagnosed with NAFLD based on histologic findings; 18 subjects were examined as controls. Therefore, 106 subjects (NASH, 70; NAFL, 18; controls, 18) were enrolled; their baseline characteristics are listed in Table 1. There were no significant differences in age, sex ratio, or BMI between NASH and NAFL patients. Regarding laboratory parameters, AST, ALT, ANA, IgG, and hyaluronic acid were significantly increased in NASH compared to NAFL patients. Regarding liver pathological findings, the degrees of LI, ballooning, and liver fibrosis were significantly higher in NASH than NAFL patients. FibroScan^®^ (FS; Echosens™, Paris, France) values were also significantly higher in NASH patients. In NAFLD patients, BMI and the levels of AST, ALT, GGT, total bilirubin, HDL-C, triglycerides, and HbA1c were significantly increased compared to controls.

**Table 1:** Clinical characteristics and pathological changes in livers of 88 NAFLD patients and 18 controls

Paramter	Controls (*n* = 18)	NAFL (*n* = 18)	NASH (*n* = 70)
Age (years)	56 (36.5–68.0)	45.5 (38.5–52.8)	57 (42.8–67.3)
Male sex (%)	66.7	50.0	38.6
BMI (kg/m2)	21.1 (18.7–22.8)	26.9 (24.4–29.8) **	28.3 (25.0–31.6) **
Albumin (g/dL)	3.9 (3.6–4.2)	4.1 (3.7–4.3)	4.1 (3.8–4.4)
AST (U/L)	16.5 (14.0–21.5)	32 (23.5–35.5) **	52 (37.8–82.0) **##
ALT (U/L)	13.5 (9.0–22.3)	43 (32.8–60.0) **	67 (50.0–109.3) **##
GGT (U/L)	14 (11.3–16.8)	67.5 (48.0–108.0) **	59 (40.3–91.0) **
Total bilirubin (mg/dL)	0.6 (0.43–0.70)	0.9 (0.60–1.25) *	0.8 (0.60–1.00) *
HDL-C (mg/dL)	60 (57.2–67.2)	50.4 (43.7–56.8) *	51.6 (41.5–58.7) *
LDL-C (mg/dL)	103 (86–117)	117 (93.5–130.0)	117 (94–135)
Triglycerides (mg/dL)	68 (52–102)	153.5 (125.0–225.3) **	111.5 (90.5–144.5) *#
Creatinine (mg/dL)	0.76 (0.57–0.95)	0.76 (0.59–0.89)	0.66 (0.56–0.81)
CRP (mg/dL)	0.09 (0.03–0.19)	0.09 (0.03–0.16)	0.11 (0.08–0.28)
Platelet count (10,000/µl)	24.4 (20.9–28.6)	22.9 (21.3–25.5)	21.6 (16.2–28.0)
Prothrombin time (%)	100 (100–100)	100 (99–100)	100 (97.5–100.0)
FBG (mg/dL)	97 (92.5–113.0)	88 (83.8–93.8)	96 (86.0–114.0)
HbA1c (N)	5.5 (5.3–5.7)	6 (5.7–7.1) *	6 (5.7–6.9) **
ANA			
Negative (%)		**15 (83.3%)**	**38 (54.3%) #**
Positive (%)		**3 (16.7%)**	**32 (45.7%)**
≧ 1:40 (%)		**3 (100%)**	**22 (68.8%)**
≧ 1:320 (%)		**0 (0%)**	**7 (21.9%)**
≧ 1:2560 (%)		**0 (0%)**	**3 (9.4%)**
AMA-M2 (index)		0.7 (0.50–0.83)	1.1 (0.5–1.7)
IgA (mg/dL)		239 (187.8–276.0)	248 (206–337)
IgG (mg/dL)		**1078 (914.5–1369.0)**	**1295.5 (1115.0–1555.8) #**
IgM (mg/dL)		80 (75.5–114.5)	96.5 (64–133)
Hyaluronic acid (ng/mL)		**12.4 (10.0–22.7)**	**45.8 (20.4–80.6) #**
Liver Stiffness (kPa)		**6 (5.3–6.8)**	**9.6 (7.3–12.0) ##**
CAP (dB/m)		291 (250.5–325.0)	306.5 (268.0–333.8)
NAS			
Steatosis			
0		0 (0%)	0 (0%)
1		10 (55.6%)	36 (51.4%)
2		5 (27.7%)	25 (35.7%)
3		3 (16.7%)	9 (12.9%)
Lobular inflammation			
0		**5 (27.8%)**	**1 (1.4%) ##**
1		**11 (61.1%)**	**34 (48.6%)**
2		**2 (11.1%)**	**34 (48.6%)**
3		**0 (0%)**	**1 (1.4%)**
Ballooning			
0		**18 (100%)**	**0 (0%) ##**
1		**0 (0%)**	**49 (70.0%)**
2		**0 (0%)**	**21 (30.0%)**
Fibrosis			
0		**7 (38.9%)**	**2 (2.9%) ##**
1		**11 (61.1%)**	**27 (38.6%)**
1A		**8 (72.7%)**	**17 (63.0%)**
1B		**3 (27.3%)**	**10 (37.0%)**
1C		**0 (0%)**	**0 (0%)**
2		**0 (0%)**	**14 (20.0%)**
3		**0 (0%)**	**24 (34.2%)**
4		**0 (0%)**	**3 (4.3%)**

Bold values indicate the parameter significantly changes between NASH and NAFL patients

^*^p < 0.05 compared with controls, **p < 0.001 compared with controls

^#^*p* < 0.05 NASH compared with NAFL, ##*p* < 0.001 NASH compared with NAFL

Values are presented as N, N (%), or median (P25–P75)

### Peripheral lymphocyte patterns of NASH in NAFLD

**Fig. 2:**
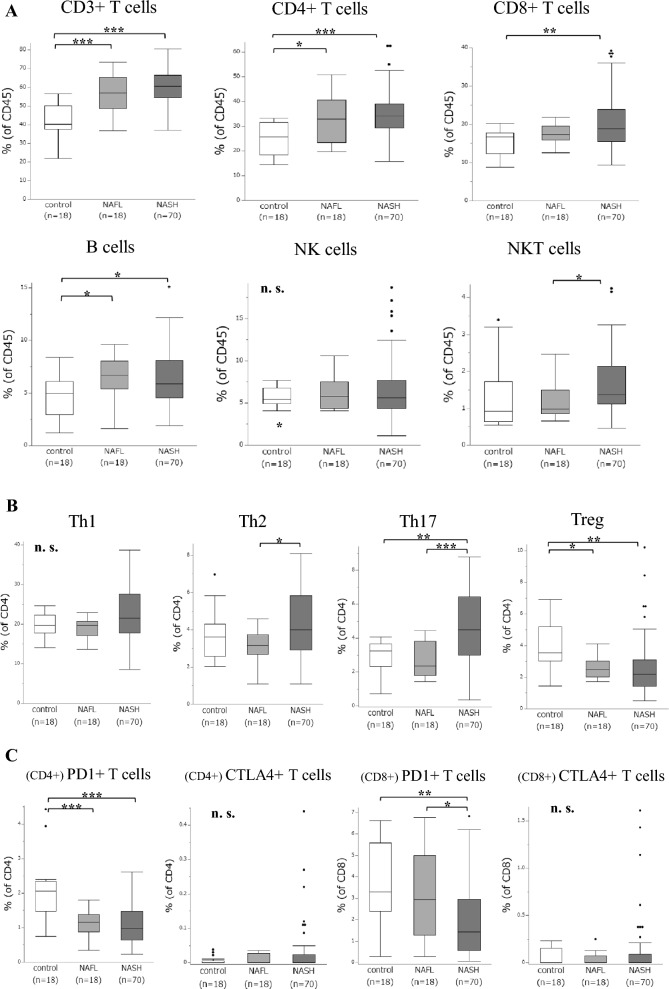
Frequencies of peripheral lymphocytes in 88 NAFLD patients and 18 controls. Gated on CD45^+^: **A** T cells, B cells, NK cells, and NKT cells. Gated on CD4^+^: **B** Th subsets. Gated on CD4^+^: (C) CD4^+^ PD1^+^ and CD4^+^ CTLA4^+^ T cells. Gated on CD8^+^: **C** CD8^+^ PD1^+^ and CD8^+^ CTLA4^+^ T cells. (*p < 0.05, **p < 0.01, ***p < 0.001; ns, not significant; Kruskal–Wallis test)

Several significant differences in lymphocyte frequencies were detected between NASH and NAFL (Fig. 2A–C). NASH patients had a significantly higher percentage of NKT cells than NAFL patients. They also had significantly higher percentages of Th2 and Th17 and a significantly lower percentage of CD8^+^ PD1^+^ T cells. Compared to controls, NAFLD patients had significantly higher percentages of CD3^+^ and CD4^+^ T cells and B cells, and a significantly lower percentage of Treg and CD4^+^ PD1^+^ T cells. These results implicate hepatic steatosis in the increased populations of peripheral T and B cells and the suppression of immune tolerance.

In univariate analyses, the levels of AST, ALT, ANA, IgG, and hyaluronic acid; liver stiffness value; and the frequencies of NKT cells, Th2, Th17, and CD8^+^ PD1^+^ T cells were significant determinants of NASH (Table 2). Multivariate analysis of these laboratory parameters and lymphocyte frequencies except ANA, IgG, and NKT cells suggested that the hyaluronic acid level, liver stiffness value, and the frequencies of Th17 and CD8^+^ PD1^+^ T cells are independent risk factors of NASH.

**Table 2:** Univariable and multivariable ORs to indicate independent risk factors of NASH in NAFLD (*n* = 88)

Parameter	Univariate	95% CI	*p*	Mutivariate		*p*
	Odds ratio			Odds ratio	95% CI	
AST (U/L)	**1.09**	**1.03–1.15**	** < 0.001**	1.13	0.97–1.33	0.074
ALT (U/L)	**1.03**	**1.01–1.05**	**0.001**	1.00	0.95–1.06	0.939
Triglycerides (mg/dL)	1.00	0.99–1.00	0.172			
ANA	**1.01**	**0.99–1.04**	**0.030**	N. A		
IgG (mg/dL)	**1.00**	**1.00–1.00**	**0.033**	N. A		
Hyaluronic acid (ng/mL)	**1.03**	**1.00–1.06**	**0.001**	**1.05**	**1.00–1.09**	**0.014**
Liver stiffness (kPa)	**1.53**	**1.14–2.06**	** < 0.001**	**1.55**	**0.94–2.55**	**0.038**
NKT cells of CD45 + cells (%)	**2.56**	**1.00–6.54**	**0.027**	N. A		
Th2 of CD4 T cells (%)	**1.76**	**1.13–2.74**	**0.004**	0.75	0.27–2.04	0.566
Th17 of CD4 + T cells (%)	**1.93**	**1.29–2.88**	** < 0.001**	**2.78**	**1.05–7.36**	**0.013**
PD1 + cells of CD8 + T cells (%)	**0.73**	**0.56–0.95**	**0.009**	**0.36**	**0.15–0.87**	**0.002**

The bold means the parameter significantly indicates risk factors of NASH in univariate or multivariate analysis

Logistic regression was used for univariate and multivariate analysis of the association between significant parameters and indicators of NASH. *NA* not assessed

### NASH patient characteristics

Seventy NASH patients (ANA negative, 36; positive, 20; AIH-overlap, 14) were enrolled; their baseline characteristics are listed in Table 3. According to the International Autoimmune Hepatitis Group (IAIHG) scores, 30–40% of ANA-negative NASH had probable AIH, all ANA-positive NASH patients had probable AIH, and all AIH-overlap NASH patients had definite AIH. Notably, two AIH-overlap NASH patients were ANA-negative. Therefore, irrespective of the presence of ANA, several NASH cases may show overlap with AIH. There were no significant differences in age and BMI between the patients with and without AIH-overlap NASH. Regarding laboratory parameters, the levels of AMA and IgM were significantly increased in AIH-overlap NASH patients. Regarding liver pathological findings, the degree of steatosis was significantly lower in AIH-overlap NASH patients. Furthermore, in ANA-positive and AIH-overlap NASH patients, the levels of ANA, AMA, and IgG were significantly increased compared to those who were ANA-negative, whereas the male sex ratio was significantly lower. Therefore, females are more likely to develop autoimmunity-related NASH compared to males.

**Table 3:** Clinical characteristics and pathological changes in the livers of 70 NASH patients

Paramter	NASH		
	ANA(−) (*n* = 36)	ANA(+) (*n* = 20)	AIH-overlap (*n* = 14)
IAIHG score			
≥ 15 (definite AIH)	0 (0%)	0 (0%)	**14 (100%) ††‡‡**
10–15 (probable AIH)	13 (36.1%)	20 (100%)	0 (0%)
< 10	23 (63.9%)	0 (0%)	0 (0%)
Age (years)	51.5 (40.8–66.3)	58.5 (53.3–67.3)	59 (39.8–68.5)
Male sex (%)	58.3	25 †	14.3 †
BMI (kg/m2)	28.7 (25.8–31.5)	28.2 (23.9–32.2)	26.9 (23.6–30.3)
Albumin (g/dL)	4.2 (3.7–4.5)	4.1 (4.0–4.2)	4.0 (3.8–4.4)
AST (U/L)	46 (36.5–70.5)	51.5 (42.8–77.3)	82 (59–87.3) †
ALT (U/L)	68.5 (47.5–108.3)	70.5 (47.5–126.0)	66 (64.5–90.5)
GGT (U/L)	58 (41.8–80.5)	59 (34.0–88.5)	61.5 (41.8–138.8)
Total bilirubin (mg/dL)	0.75 (0.60–1.00)	0.8 (0.70–1.10)	0.85 (0.68–0.93)
HDL-C (mg/dL)	46.0 (40.4–53.5)	57.7 (47.6–69.5) †	53.8 (49.0–58.5)
LDL-C (mg/dL)	119 (97–135)	109.5 (95.0–127.3)	100.5 (88.0–153.0)
Triglycerides (mg/dL)	116 (90.5–175.0)	103 (74.3–134.8)	117 (110.5–127.3)
Creatinine (mg/dL)	0.72 (0.56–0.87)	0.69 (0.57–0.81)	0.57 (0.56–0.60)
CRP (mg/dL)	0.12 (0.10–0.28)	0.10 (0.07–0.26)	0.12 (0.07–0.51)
Platelet count (10,000/µl)	21.6 (17.3–28.0)	21.1 (20.1–27.7)	15.6 (14.4–20.7)
Prothrombin time (%)	100 (94.5–100.0)	100 (100–100)	100 (100–100)
FBG (mg/dL)	97 (86.3–115.5)	98 (86.5–107.5)	91 (81.0–104.5)
HbA1c (N)	6.1 (5.6–7.2)	6.1 (5.9–6.9)	6 (5.5–6.1)
ANA			
Negative (%)	36 (100%)	0 (0%) ††	2 (14.2%) ††
Positive (%)	0 (0%)	20 (100%)	12 (85.8%)
≧ 1:40 (%)	0 (0%)	17 (85%)	5 (41.7%)
≧ 1:320 (%)	0 (0%)	3 (15%)	4 (33.3%)
≧ 1:2560 (%)	0 (0%)	0 (0%)	3 (25%)
AMA-M2 (index)	0.55 (0.50–0.95)	1.4 (0.8–1.7) †	**1.7 (1.65–2.20) ††‡**
IgA (mg/dL)	230.5 (202.0–306.5)	280.5 (233.0–356.3)	255 (214.5–451.5)
IgG (mg/dL)	1189 (1049.0–1338.5)	1360 (1159.0–1575.5) †	1611 (1429.8–1922.3) ††
IgM (mg/dL)	80.5 (57.8–111.0)	93 (58.3–139.3)	**133 (121.8–153.0) ††‡**
Hyaluronic acid (ng/mL)	37.5 (11.9–60.1)	50.2 (26.4–82.1)	109.8 (66.1–152.0) †
Liver Stiffness (kPa)	10.2 (8.5–13.2)	8.1 (6.8–10.3)	10 (6.2–15.9)
CAP (dB/m)	306.5 (274.8–338.5)	313.5 (273.3–342.0)	**257 (203.8–319.5) †‡**
NAS			
Steatosis			
0	0 (0%)	0 (0%)	0 (0%) **†‡**
1	18 (50%)	8 (40%)	10 (71.4%)
2	11 (30.6%)	10 (50%)	4 (28.6%)
3	7 (19.4%)	2 (10%)	0 (0%)
Lobular inflammation			
0	1 (2.8%)	0 (0%)	0 (0%)
1	19 (52.7%)	10 (50%)	5 (35.7%)
2	15 (41.7%)	10 (50%)	9 (64.3%)
3	1 (2.8%)	0 (0%)	0 (0%)
Ballooning			
0	0 (0%)	0 (0%)	0 (0%)
1	26 (72.2%)	15 (75%)	8 (57.1%)
2	10 (27.8%)	5 (25%)	6 (42.9%)
Fibrosis			
0	0 (0%)	2 (10%)	0 (0%) ‡
1	17 (47.2%)	9 (45%)	1 (7.2%)
1A	13 (76.5%)	4 (20%)	0 (0%)
1B	4 (23.5%)	5 (25%)	1 (7.2%)
1C	0 (0%)	0 (0%)	0 (0%)
2	5 (13.9%)	6 (30%)	3 (21.4%)
3	11 (30.6%)	3 (15%)	10 (71.4%)
4	3 (8.3%)	0 (0%)	0 (0%)

Bold values indicate the parameter significantly changes between AIH-overlap and ANA -/+ NASH patients

^†^*p* < 0.05 compared with ANA(−), ††*p* < 0.001 compared with ANA(-)

^‡^*p* < 0.05 AIH-overlap compared with ANA( +), ‡‡*p* < 0.001 AIH-overlap compared with ANA( +)

Values are presented as N, N (%), or median (P25–P75)

### Peripheral lymphocyte patterns of AIH-overlap NASH in NASH

**Fig. 3:**
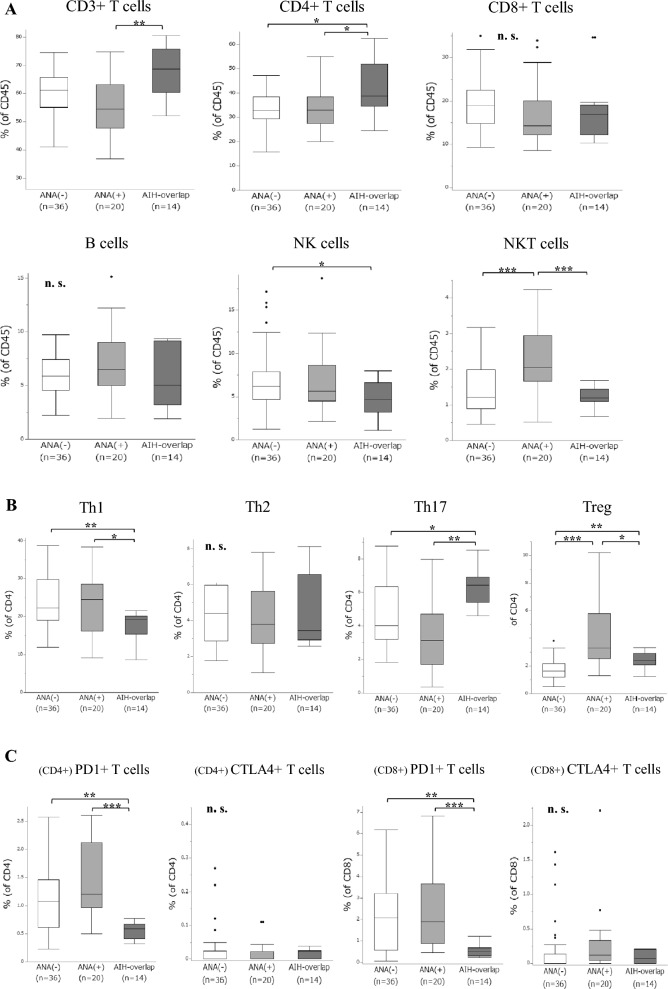
Frequencies of peripheral lymphocytes in 70 NASH patients. Gated on CD45^+^: **A** T cells, B cells, NK cells, and NKT cells. Gated on CD4^+^: **B** Th subsets. Gated on CD4^+^: **C** CD4^+^ PD1^+^ and CD4^+^ CTLA4^+^ T cells. Gated on CD8^+^: **C** CD8^+^ PD1^+^ and CD8^+^ CTLA4^+^ T cells. (*p < 0.05, **p < 0.01, ***p < 0.001; *ns* not significant; Kruskal–Wallis test)

Several significant differences in lymphocyte frequencies were observed between patients with and without AIH-overlap NASH (Fig. 3A–C). AIH-overlap NASH patients had significantly higher percentages of CD4^+^ T cells and Th17, and significantly lower percentages of Th1, CD4^+^ and CD8^+^ PD1^+^ T cells. Compared to ANA-negative NASH patients, those who were ANA-positive with AIH-overlap showed a significantly higher percentage of Tregs, suggesting that autoimmunity-related NASH may induce peripheral Tregs.

Furthermore, the changes in peripheral lymphocyte frequencies of 70 NASH patients according to the degree of NAS, fibrosis, and the levels of AST, ALT, and IgG were examined to show the associations between these lymphocytes and AIH-overlap NASH (Supplementary Table 2). Regarding liver damage, the frequency of CD4^+^ T cells was positively correlated with the levels of AST and ALT, while that of CD8^+^ T cells was negatively correlated. Notably, regarding liver fibrosis, the frequency of CD8^+^ PD1^+^ T cells was positively correlated with the degree of fibrosis, while that of NKT cells was negatively correlated; that of CD8^+^ PD1^+^ T cells was also negatively correlated with the IgG level. These results suggest that differential peripheral lymphocyte subsets are deeply associated with NASH; among them, CD8^+^ PD1^+^ T cells may be associated with liver fibrosis and autoimmunity in NASH.

### Risk factors of AIH-overlap NASH in NASH

In univariate analyses, male sex; the levels of ANA, IgG, and IgM; CAP value; and frequencies of CD4^+^ T cells, NK cells, Th1, Th17, CD4^+^ and CD8^+^ PD1^+^ T cells were significant determinants of AIH-overlap NASH (Table 4). Multivariate analysis of these laboratory parameters and lymphocyte frequencies except male sex, CD4^+^ T cells, and NK cells suggested that the frequency of CD8^+^ PD1^+^ T cells is an independent risk factor of AIH-overlap NASH. Next, we examined the changes in several laboratory parameters and peripheral lymphocyte frequencies, including CD8^+^ PD1^+^ T cells, before and after steroid therapy for 9 AIH-overlap NASH patients (Supplementary Table 3). The decreased frequency of CD8^+^ PD1^+^ T cells was significantly increased by steroid treatment, which may increase the usefulness of the frequency of CD8^+^ PD1^+^ T cells as an indicator of AIH-overlap NASH.

**Table 4:** Univariable and multivariable ORs to indicate independent risk factors of AIH overlapping NASH in NASH (*n *= 70)

Parameter	Univariate	95% CI	*p*	Mutivariate		*p*
	Odds Ratio			Odds Ratio	95% CI	
Male sex (%)	**0.19**	**0.04–0.94**	**0.031**	N. A		
ANA	**1.01**	**1.00–1.02**	** < 0.001**	1.00	0.99–1.01	0.688
AMA-M2 (index)	1.12	0.97–1.29	0.107			
IgG (mg/dL)	**1.00**	**1.00–1.01**	**0.003**	1.00	0.99–1.01	0.540
IgM (mg/dL)	**1.03**	**1.01–1.04**	**0.001**	1.06	0.95–1.20	0.126
CAP (dB/m)	**0.98**	**0.96–0.99**	**0.002**	0.94	0.84–1.04	0.052
CD4 + T cells of CD45 + cells (%)	**1.12**	**1.03–1.19**	**0.022**	N. A		
NK cells of CD45 + cells (%)	**0.76**	**0.58–1.01**	**0.025**	N. A		
Th1 of CD4 + T cells (%)	**0.86**	**0.77–0.96**	**0.006**	1.55	0.61–3.93	0.138
Th17 of CD4 + T cells (%)	**1.65**	**1.11–2.443**	**0.005**	0.35	0.06–2.28	0.130
Treg of CD4 + T cells (%)	0.93	0.63–1.38	0.707			
PD1 + cells of CD4 + T cells (%)	**0.01**	**0.00–0.23**	** < 0.001**	0.01	0.00–99.1	0.236
PD1 + cells of CD8 + T cells (%)	**0.11**	**0.02–0.67**	** < 0.001**	**0.01**	**0.00–38.9**	**0.004**

Bold values indicate the parameter significantly indicates risk factors of AIH-overlap NASH in univariate or multivariate analysis

Logistic regression was used for univariate and multivariate analysis of the association between significant parameters and indicators of NASH. *N. A*. not assessed

Furthermore, the target lymphocyte frequencies in 27 PBMC samples of AIH patients (acute:chronic = 12:15) were evaluated. These frequencies and baseline characteristics of total 41 patients together with 14 AIH-overlap NASH patients are listed in Supplementary Table 4. AIH-overlap NASH patients had a significantly lower percentage of CD4^+^ and CD8^+^ PD1^+^ T cells compared to those with acute and chronic AIH; there were no significant differences in autoimmunity-related parameters (ANA, AMA-M2, IgA, IgM, and Tregs). These results suggest that PD1 expression differs between AIH-overlap NASH and AIH, although these two diseases share several autoimmunity-related parameters.

## Discussion

We examined differential patterns of peripheral lymphocytes between NAFLD and AIH patients. NAFLD and AIH reportedly show different peripheral lymphocyte patterns [21, 42]; however, to the best of our knowledge, the patterns in NASH with autoimmunity are unknown. Selection of NASH cases suspected to overlap with AIH prior to pathological diagnosis is clinically vital. In the present cohort, only a decreased frequency of peripheral CD8^+^ PD1^+^ T cells was an independent risk factor of AIH-overlap NASH in univariate and multivariate analyses of peripheral lymphocyte frequencies and clinical parameters associated with NAFLD and AIH. The frequencies of CD4^+^ and CD8^+^ PD1^+^ T cells were decreased in NASH compared to NAFL, and in AIH-overlap NASH compared to AIH alone. These findings suggest that immune tolerance centered on PD1 is impaired in peripheral blood of NASH patients; PD1 expression in peripheral CD8^+^ T cells was lower in AIH overlap.

PD1, typically expressed on activated CD4^+^ and CD8^+^ T cells, is an important regulator of immune homeostasis and tolerance, and suppresses T cell proliferation [20, 43]. In NAFLD, PD1 expression in peripheral CD4^+^ T cells is decreased and activated CD4^+^ T cells are not dysregulated [17]; our results reflected these findings (Fig. 2C). CD8^+^ T cells are important for the protective immunity against chronic infections and cancer; however, persistent antigen stimulation results in T cell exhaustion, and exhausted CD8^+^ T cells have decreased effector function and proliferative capacity, caused by PD1 overexpression [43]. In chronic liver damage, as shown in Supplementary Table 2, peripheral CD8^+^ T cells may be decreased by their exhaustion, while CD4^+^ T cells may be increased by the production of proinflammatory cytokines [44, 45]. In NAFLD, PD1 expression in peripheral CD8^+^ T cells is significantly increased in NASH model mice compared to control mice, and potentially limits T-cell mediated liver damage in murine steatohepatitis [34, 46]. Conversely, in this study, the frequency of peripheral CD8^+^ PD1^+^ T cells was decreased in NASH compared to non-NASH patients. Furthermore, the frequency of PD1^+^ T cells is significantly decreased in vitro by treatment with fatty acids that increase in NAFLD/NASH patients [17]; the decrease in PD1 expression due to the increased fatty acid may be related to impaired lipid metabolism, which might be the reason why the frequency of peripheral CD8^+^ PD1^+^ T cells was decreased in NASH despite the state of immune activation. PD1 expression in peripheral T cells in NASH patients may be dissimilar to NASH animal models and be decreased by impaired lipid metabolism. Peripheral CD8^+^ PD1^+^ T cells may have indicative power for NASH in NAFLD.

In autoimmunity, PD1 is involved in rheostatic modulation of T cell receptor (TCR) signaling that prevents the development of autoreactive T cells and the following tissue destruction, and PD1 expression in CD4^+^ and CD8^+^ T cells is increased [40, 47]. In AIH patients, PD1 expression in peripheral CD4^+^ T cells is significantly increased compared to healthy controls, and there are no significant differences in peripheral CD8^+^ T cells [48]; Our results reflected these findings (Supplementary Table 5). CD4^+^ T cells are essential for induction of AIH, whereas CD8^+^ T cells are linked to progression to fatal hepatic damage [47]; in autoimmunity, where evidence of CD4^+^ T-cell co-stimulation is remarkable, that of CD8^+^ T-cell exhaustion is reduced [49]. In this study, the frequency of peripheral CD4^+^ T cells in AIH-overlap NASH was significantly increased compared to the other types of NASH, and no significant differences in peripheral CD8^+^ T cells were observed. Regarding PD1 expression between AIH and NASH, both frequencies of peripheral CD4^+^ and CD8^+^ PD1^+^ T cells in AIH-overlap NASH were decreased compared to the other types of NASH and significantly recovered due to improvement of AIH by steroid treatment. The reason is unclear; however, rheostatic modulation of TCR signaling in autoimmunity may be dysregulated by the increased fatty acid and impaired lipid metabolism in NASH, and therefore, immune tolerance may be dysregulated in patients with AIH and NASH. Furthermore, hepatic fibrosis is the most important prognostic factor in NAFLD/NASH [50]; in this study, as shown in Supplementary Table 2, CD8^+^ PD1^+^ T cells were associated with liver fibrosis, not CD4^+^ PD1^+^ T cells. CD8^+^ T cells in the liver are associated with liver fibrosis, which may be true of exhausted peripheral CD8^+^ T cells by PD1 overexpression [43, 51]. Overall, in NAFLD, peripheral CD8^+^ PD1^+^ T cells may be more indicative of AIH-overlap NASH than CD4^+^ PD1^+^ T cells.

Serum levels of IgM and AMA in AIH-overlap NASH were significantly increased compared to other types of NASH. These levels are significantly increased in autoimmune diseases [26, 52], which may be true of AIH-overlap NASH and AIH alone (Table 3 and Supplementary Table 4). They are also significantly increased in PBC patients [27]. In fact, there are several PBC patients with features of AIH [53], which suggests that PBC may not be completely ruled out among AIH patients; therefore, these two levels may not be useful for distinguishing AIH-overlap NASH from other types of NASH.

Serum levels of hyaluronic acid, liver stiffness, and frequencies of Th17 cells were independent risk factors of NASH. Hyaluronic acid, a serum fibrosis marker, is useful for assessing NAFLD-related fibrosis and shows promise as a noninvasive biomarker of NASH [12]. However, the level can be increased by dietary factors or autoimmune diseases [54]. Liver stiffness value measured by elastography is also useful for assessing NAFLD-related fibrosis and is more accurate than serum markers for fibrosis assessment; however, it is unreliable in morbidly obese individuals [55]. Th17 cells are important in the inflammatory processes characterizing NASH [56, 57]. The peripheral Th17/Treg ratio is significantly increased in NASH compared to NAFL, and maintains intestinal barrier integrity in NAFLD [56]; peripheral Th17 cells may be indicative of NASH. Tregs are reciprocally associated with Th17 cells in the outcome of immune responses, particularly in autoimmune diseases [58], and are important in immune tolerance in addition to PD1 [21, 40]. In this study, peripheral Tregs in NAFLD and AIH patients were significantly decreased compared to controls (Fig. 2 and Supplementary Table 5); therefore, peripheral Tregs in NAFLD may reflect hepatic autoimmunity and lipid metabolism or steatosis.

Peripheral NK, NKT, and Th1 cells may be associated with NASH pathogenesis and autoimmunity. NK cells mediate important hepatoprotective functions in chronic liver disease [59]; however, their functions in NAFLD are unknown. Peripheral NK cell numbers in NASH are not altered compared to non-NASH, and peripheral NK cell numbers in AIH decrease with increasing hepatic accumulation of NK cells [42, 60]. Our findings were similar, and peripheral NK cells in AIH-overlap NASH may have few hepatoprotective effects. NKT cells have anti-inflammatory functions and attenuate adiposity, inflammation, and insulin resistance in NAFLD [61]. In the human liver, accumulation of invariant NKT cells is associated with liver disease progression, particularly NASH-related LC [62]. Peripheral NKT cell numbers in NASH are significantly increased compared to non-NASH [35], and vary depending on the type of AIH [57, 63]. In this study, AIH-overlap NASH showed a significantly lower percentage of peripheral NKT cells compared to ANA-positive NASH, and there were no significant differences between AIH-overlap NASH and ANA-negative NASH. Therefore, peripheral NKT cell numbers in NASH may vary depending on the presence of autoimmunity. Th1 cells are involved in adipose tissue inflammation associated with obesity-related pathologies [57]. No consensus has been reached on whether peripheral their numbers increase or decrease in NASH [18]; their numbers in AIH are significantly increased compared to healthy individuals [64]. In our study, AIH-overlap NASH had a significantly lower percentage of these compared to the other types of NASH and chronic AIH; therefore, the role of peripheral Th1 cells in autoimmunity may differ between AIH-overlap NASH and chronic AIH.

This study had several limitations. First, peripheral lymphocyte patterns examined do not directly reflect hepatic immunokinetics. The frequencies of several lymphocyte subsets differ between peripheral blood and liver [59, 63]. In this study, FCM of liver biopsies and validation of the frequency of CD8^+^ PD1^+^ T cells, an independent risk factor of AIH-overlap NASH, in another cohort of PBMCs could not be performed; therefore, examining the frequency in the liver and another cohort will be a future task. Second, the numbers of NASH and NAFL patients differed. In our hospital, patients with NAFLD suspicious for NASH including overlap with AIH are recommended to undergo invasive liver biopsy, increasing the number of NASH patients in the cohort. Third, no definite criteria have been established for diagnosing AIH-overlap NASH [4, 7, 65], and the establishment of diagnostic procedures for AIH-overlap NASH will be needed in the future. Finally, patients with LC and HCC plus NASH were excluded. Information on the peripheral lymphocyte patterns of these patients will enable the prevention of LC and HCC.

In conclusion, the frequencies of peripheral lymphocytes differed between AIH-overlap NASH and other types of NASH. In univariate and multivariate analyses, only the frequency of CD8^+^ PD1^+^ T cells was an independent risk factor of NASH overlapping with AIH in the present cohort. Our data suggest that pathological diagnosis of AIH-overlap NASH by liver biopsy is warranted for NASH cases with significantly decreased frequency of peripheral CD8^+^ PD1^+^ T cells. Finally, our FCM method will represent a novel means of indicating risk factors of NASH with autoimmunity, in addition to biochemical, imaging, and pathological tests.

### Supplementary Information

Below is the link to the electronic supplementary material.

Supplementary file1 Fig. 1 Flow chart of the study population. A total of 133 PBMC samples was obtained from 115 NAFLD and AIH patients and 18 controls. Liver biopsy was performed on the 115 patients at the University of Tokyo; not on controls. Among the patients, 88 were diagnosed with NAFLD, 27 with AIH (acute:chronic = 12:15). Regarding NAFLD, 70 patients were diagnosed as NASH, 18 as NAFL. The 70 NASH patients were divided into three groups: ANA negative, 36; ANA positive, 20; and AIH-overlap, 14., FCM plots and gating strategies used to identify PD1+ T cells: (A) FMO for CD4+ PD1+ (left) and CD8+ PD1+ T cells (right); (B) gated on CD4+ PD1+ (left) and CD8+ PD1+ T cells (right). Data are from one healthy control. (PPTX 115 KB)

Supplementary file2 (DOCX 28 KB)

Supplementary file3 (DOCX 48 KB)

## Data Availability

The data of this study are available on request from the corresponding author. The data are not publicly available due to privacy reasons.

## Abbreviations

NAFLD: Nonalcoholic fatty liver disease
NASH: Nonalcoholic steatohepatitis
LC: Liver cirrhosis
HCC: Hepatocellular carcinoma
ANA: Antinuclear antibody
AIH: Autoimmune hepatitis
PBMC: Peripheral blood mononuclear cells
NK: Natural killer
FCM: Flow cytometry
NAFL: Nonalcoholic fatty liver
AST: Aspartate aminotransferase
ALT: Alanine aminotransferase
PBC: Primary biliary cholangitis
DILI: Drug-induced liver injury
AUS: Abdominal ultrasonography
Ig: Immunoglobulin
BMI: Body mass index
GGT: Gamma-glutamyl transpeptidase
HDL: High-density lipoprotein
LDL: Low-density lipoprotein
CRP: C-reactive protein
FBG: Fasting blood glucose
HbA1c: Hemoglobin A1c
AMA: Anti-mitochondrial
AILD: Autoimmune liver disease
CAP: Controlled attenuation parameter
FS: FibroScan
NAS: NAFLD activity score
LI: Lobular inflammation
IAIHG: International autoimmune hepatitis group
FACS: Fluorescence-activated cell sorting
Th: Helper T cell
CXCR: C–X–C chemokine receptor
CCR: C–C chemokine receptor
Treg: Regulatory T cells
PD: Programmed cell death
CTLA: Cytotoxic T-lymphocyte antigen
TCR: T cell receptor

## References

[1] Anstee QM, Targher G, Day CP (2013). Progression of NAFLD to diabetes mellitus, cardiovascular disease, or cirrhosis. Nat Rev Gastroenterol Hepatol.

[2] Tokushige K, Hyogo H, Nakajima T (2016). Hepatocellular carcinoma in Japanese patients with nonalcoholic fatty liver disease and alcoholic liver disease: multicenter survey. J Gastroenterol.

[3] Lonardo A, Nascimbeni F, Mantovani A (2018). Hypertension, diabetes, atherosclerosis, and NASH: Cause or consequence?. J Hepatol.

[4] Niwa H, Sasaki M, Haratake J (2007). Clinicopathological significance of antinuclear antibodies in non-alcoholic steatohepatitis. Hepatol Res.

[5] Adams LA, Lindor KD, Angulo P (2004). The prevalence of autoantibodies and autoimmune hepatitis in patients with nonalcoholic fatty liver disease. Am J Gastroenterol.

[6] Woods CP, Hazlehurst JM, Tomlinson JW (2015). Glucocorticoids and non-alcoholic fatty liver disease. J Steroid Biochem Mol Biol.

[7] Komura T, Ohta H, Seike T (2018). The efficacy of corticosteroid therapy in a patient with non-alcoholic steatohepatitis overlapping autoimmune hepatitis. Intern Med.

[8] Chalasani N, Younossi Z, Lavine JE (2012). The diagnosis and management of non-alcoholic fatty liver disease: practice guideline by the American association for the study of liver diseases, American college of gastroenterology, and the American gastroenterological association. Hepatology.

[9] Sutti S, Albano E (2020). Adaptive immunity: an emerging player in the progression of NAFLD. Nat Rev Gastroenterol Hepatol.

[10] Huby T, Gautier EL (2022). Immune cell-mediated features of non-alcoholic steatohepatitis. Nat Rev Immunol.

[11] Younes R, Govaere O, Petta S (2020). Presence of serum antinuclear antibodies does not impact long-term outcomes in nonalcoholic fatty liver disease. Am J Gastroenterol.

[12] Kado A, Tsutsumi T, Enooku K (2019). Noninvasive diagnostic criteria for nonalcoholic steatohepatitis based on gene expression levels in peripheral blood mononuclear cells. J Gastroenterol.

[13] Ajmera V, Perito ER, Bass NM (2017). NASH clinical research network. novel plasma biomarkers associated with liver disease severity in adults with nonalcoholic fatty liver disease. Hepatology.

[14] Tamaki N, Kurosaki M, Huang DQ (2022). Noninvasive assessment of liver fibrosis and its clinical significance in nonalcoholic fatty liver disease. Hepatol Res.

[15] Kamada Y, Nakamura T, Isobe S (2023). JANIT forum SWOT analysis of noninvasive tests for diagnosing NAFLD with severe fibrosis: an expert review by the JANIT forum. J Gastroenterol.

[16] Ugarte-Gil MF, Sánchez-Zúñiga C, Gamboa-Cárdenas RV (2016). Circulating CD4^+^CD28null and extra-thymic CD4^+^CD8^+^ double positive T cells are independently associated with disease damage in systemic lupus erythematosus patients. Lupus.

[17] Seike T, Mizukoshi E, Yamada K (2020). Fatty acid-driven modifications in T-cell profiles in non-alcoholic fatty liver disease patients. J Gastroenterol.

[18] Lin SZ, Fan JG (2022). Peripheral immune cells in NAFLD patients: a spyhole to disease progression. EBioMedicine.

[19] Renand A, Cervera-Marzal I, Gil L (2020). Integrative molecular profiling of autoreactive CD4 T cells in autoimmune hepatitis. J Hepatol.

[20] Cueto-Sanchez A, Niu H, Del Campo-Herrera E (2021). Lymphocyte profile and immune checkpoint expression in drug-induced liver injury: An immunophenotyping study. Clin Pharmacol Ther.

[21] Peiseler M, Sebode M, Franke B (2012). FOXP3^+^ regulatory T cells in autoimmune hepatitis are fully functional and not reduced in frequency. J Hepatol.

[22] Vonghia L, Magrone T, Verrijken A (2015). Peripheral and hepatic vein cytokine levels in correlation with non-alcoholic fatty liver disease (NAFLD)-related metabolic, histological, and haemodynamic features. PLoS ONE.

[23] Strzepka J, Schwartz BA, Ritz EM, et al. Patients With Autoimmune Hepatitis and Nonalcoholic Fatty Liver Disease: Characteristics, Treatment, and Outcomes. J Clin Gastroenterol. 2022. 10.1097/MCG.0000000000001817 36729430

[24] Abe K, Fujita M, Hayashi M (2020). Association of serum 25-hydroxyvitamin D levels with severe necroinflammatory activity and inflammatory cytokine production in type I autoimmune hepatitis. PLoS ONE.

[25] European Association for the Study of the Liver (2015). EASL clinical practice guidelines: autoimmune hepatitis. J Hepatol.

[26] Takahashi A, Arinaga-Hino T, Ohira H, et al (2017); Autoimmune Hepatitis Study Group-Subgroup of the Intractable Hepato-Biliary Disease Study Group in Japan. Autoimmune hepatitis in Japan: trends in a nationwide survey. J Gastroenterol 52: 631–640. 10.1007/s00535-016-1267-0 27722997

[27] Hu S, Zhao F, Wang Q (2014). The accuracy of the anti-mitochondrial antibody and the M2 subtype test for diagnosis of primary biliary cirrhosis: A meta-analysis. Clin Chem Lab Med.

[28] Matteoni CA, Younossi ZM, Gramlich T (1999). Nonalcoholic fatty liver disease: a spectrum of clinical and pathological severity. Gastroenterology.

[29] Kleiner DE, Brunt EM, Wilson LA, et al; Nonalcoholic Steatohepatitis Clinical Research Network. Association of histologic disease activity with progression of nonalcoholic fatty liver disease. JAMA Netw Open. 2019; 2: e1912565. 10.1001/jamanetworkopen.2019.12565 PMC6784786 31584681

[30] Alvarez F, Berg PA, Bianchi FB (1999). International autoimmune hepatitis group report: review of criteria for diagnosis of autoimmune hepatitis. J Hepatol.

[31] Tiniakos DG, Brain JG, Bury YA (2015). Role of histopathology in autoimmune hepatitis. Dig Dis.

[32] Tanaka A, Notohara K (2021). Immunoglobulin G4 (IgG4)-related autoimmune hepatitis and IgG4-hepatopathy: a histopathological and clinical perspective. Hepatol Res.

[33] Tsuneyama K, Baba H, Kikuchi K (2013). Autoimmune features in metabolic liver disease: a single-center experience and review of the literature. Clin Rev Allergy Immunol.

[34] Hansel C, Erschfeld S, Baues M (2019). The inhibitory *T* cell receptors PD1 and 2B4 are differentially regulated on CD4 and CD8 T cells in a mouse model of non-alcoholic steatohepatitis. Front Pharmacol.

[35] Bergeron M, Nicholson JK, Phaneuf S (2002). Selection of lymphocyte gating protocol has an impact on the level of reliability of T-cell subsets in aging specimens. Cytometry.

[36] Huang LQ, Wang JX, He K (2018). Analysis of peripheral blood T-cell subsets and regulatory T-cells in multiple myeloma patients. Cell Mol Biol.

[37] Raphael I, Nalawade S, Eagar TN (2015). T cell subsets and their signature cytokines in autoimmune and inflammatory diseases. Cytokine.

[38] Wang SR, Zhong N, Zhang XM (2021). OMIP 071: A 31-parameter flow cytometry panel for in-depth immunophenotyping of human T-cell subsets using surface markers. Cytometry A.

[39] Wang H, Feng X, Yan W (2020). Regulatory T cells in autoimmune hepatitis: Unveiling their roles in mouse models and patients. Front Immunol.

[40] Zhang B, Chikuma S, Hori S (2016). Nonoverlapping roles of PD-1 and FoxP3 in maintaining immune tolerance in a novel autoimmune pancreatitis mouse model. Proc Natl Acad Sci U S A.

[41] Pardoll DM (2012). The blockade of immune checkpoints in cancer immunotherapy. Nat Rev Cancer.

[42] Stiglund N, Strand K, Cornillet M (2019). Retained NK cell phenotype and functionality in non-alcoholic fatty liver disease. Front Immunol.

[43] Hashimoto M, Kamphorst AO, Im SJ (2018). CD8 T cell exhaustion in chronic infection and cancer: opportunities for interventions. Annu Rev Med.

[44] Wang H, Luo H, Wan X (2020). TNF-α/IFN-γ profile of HBV-specific CD4 T cells is associated with liver damage and viral clearance in chronic HBV infection. J Hepatol.

[45] Shen XH, Xu P, Yu X (2018). Discrepant clinical significance of CD28+CD8- and CD4+CD25high regulatory T cells during the progression of hepatitis B virus infection. Viral Immunol.

[46] Penna A, Pilli M, Zerbini A (2007). Dysfunction and functional restoration of HCV-specific CD8 responses in chronic hepatitis C virus infection. Hepatology.

[47] Kido M, Watanabe N, Okazaki T (2008). Fatal autoimmune hepatitis induced by concurrent loss of naturally arising regulatory T cells and PD-1-mediated signaling. Gastroenterology.

[48] Aarslev K, Dige A, Greisen SR (2017). Soluble programmed death-1 levels are associated with disease activity and treatment response in patients with autoimmune hepatitis. Scand J Gastroenterol.

[49] McKinney EF, Lee JC, Jayne DR (2015). T-cell exhaustion, co-stimulation and clinical outcome in autoimmunity and infection. Nature.

[50] Tokushige K, Ikejima K, Ono M (2021). Evidence-based clinical practice guidelines for nonalcoholic fatty liver disease/nonalcoholic steatohepatitis 2020. J Gastroenterol.

[51] Koda Y, Teratani T, Chu PS (2021). CD8+ tissue-resident memory T cells promote liver fibrosis resolution by inducing apoptosis of hepatic stellate cells. Nat Commun.

[52] Boes M, Schmidt T, Linkemann K (2000). Accelerated development of IgG autoantibodies and autoimmune disease in the absence of secreted IgM. Proc Natl Acad Sci USA.

[53] Yoshioka Y, Taniai M, Hashimoto E (2014). Clinical profile of primary biliary cirrhosis with features of autoimmune hepatitis: Importance of corticosteroid therapy. Hepatol Res.

[54] Suarez-Fueyo A, Tsokos MG, Kwok SK (2019). Hyaluronic acid synthesis contributes to tissue damage in systemic lupus erythematosus. Front Immunol.

[55] Adams LA, Chan WK (2020). Noninvasive tests in the assessment of NASH and NAFLD Fibrosis: Now and into the future. Semin Liver Dis.

[56] Rau M, Schilling AK, Meertens J (2016). Progression from nonalcoholic fatty liver to nonalcoholic steatohepatitis is marked by a higher frequency of Th17 cells in the liver and an increased Th17/resting regulatory T cell ratio in peripheral blood and in the liver. J Immunol.

[57] Van Herck MA, Weyler J, Kwanten WJ (2019). The differential roles of T cells in non-alcoholic fatty liver disease and obesity. Front Immunol.

[58] Abe M, Hiasa Y, Onji M (2013). T helper 17 cells in autoimmune liver diseases. Clin Dev Immunol.

[59] Heymann F, Tacke F (2016). Immunology in the liver—from homeostasis to disease. Nat Rev Gastroenterol Hepatol.

[60] Xiao F, Ai G, Yan W (2018). Intrahepatic recruitment of cytotoxic NK cells contributes to autoimmune hepatitis progression. Cell Immunol.

[61] Lynch L, Nowak M, Varghese B (2012). Adipose tissue invariant NKT cells protect against diet-induced obesity and metabolic disorder through regulatory cytokine production. Immunity.

[62] Syn WK, Oo YH, Pereira TA (2010). Accumulation of natural killer T cells in progressive nonalcoholic fatty liver disease. Hepatology.

[63] Sebode M, Wigger J, Filpe P (2019). Inflammatory phenotype of intrahepatic sulfatide-reactive type II NKT cells in humans with autoimmune hepatitis. Front Immunol.

[64] Bovensiepen CS, Schakat M, Sebode M (2019). TNF-Producing Th1 cells are selectively expanded in liver infiltrates of patients with autoimmune hepatitis. J Immunol.

[65] Dalekos GN, Gatselis NK, Zachou K (2020). NAFLD and autoimmune hepatitis: do not judge a book by its cover. Eur J Intern Med.

