# Supplementary figures and images for: Noninvasive approach to indicate risk factors of nonalcoholic steatohepatitis overlapping autoimmune hepatitis based on peripheral lymphocyte pattern
Source: J Gastroenterol. 2023 Sep 14;58(12):1237–51. doi: 10.1007/s00535-023-02038-y (PMC10657798; doi:10.1007/s00535-023-02038-y)

## Slide 1
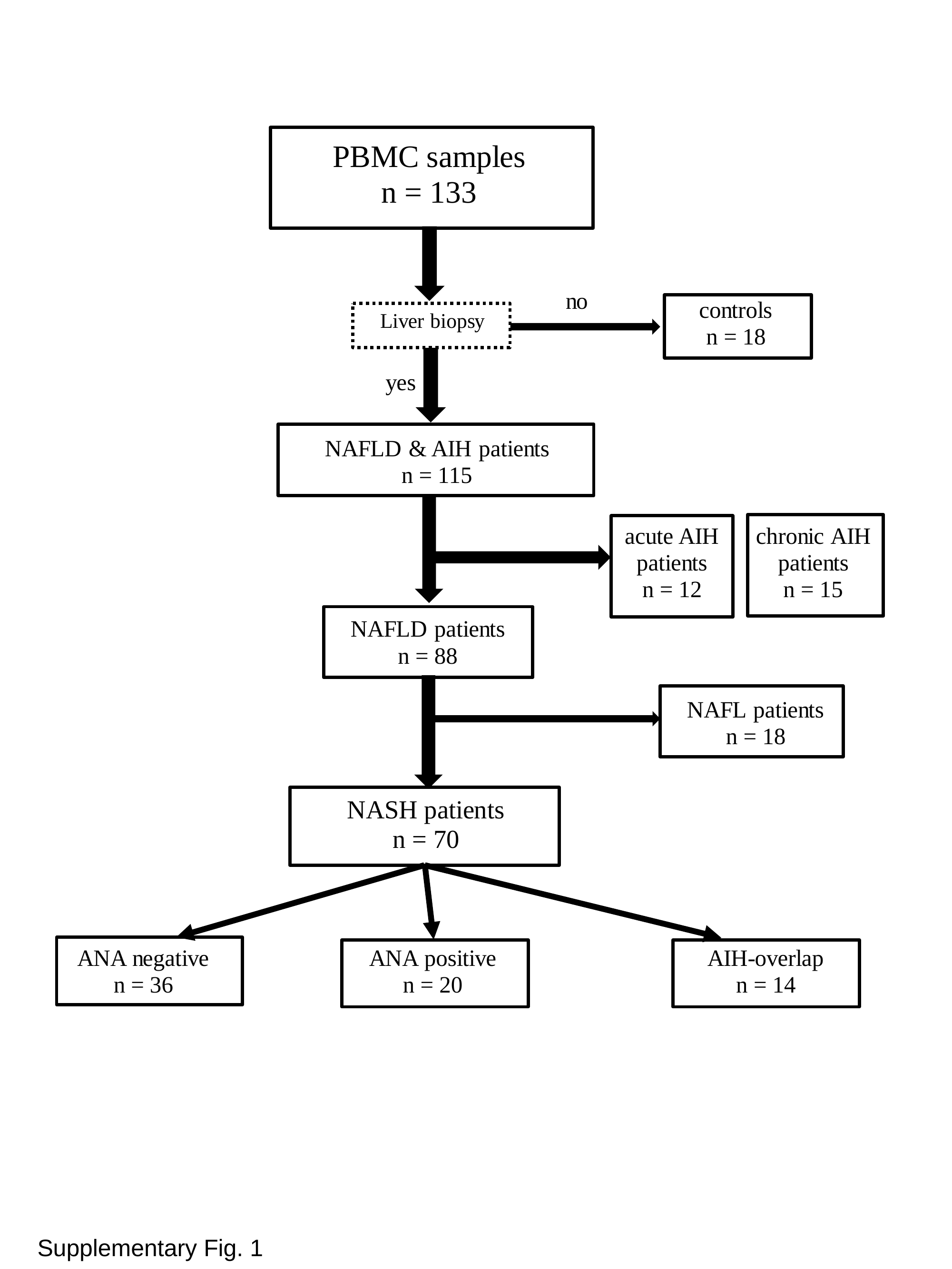

Supplementary Fig. 1

## Slide 2
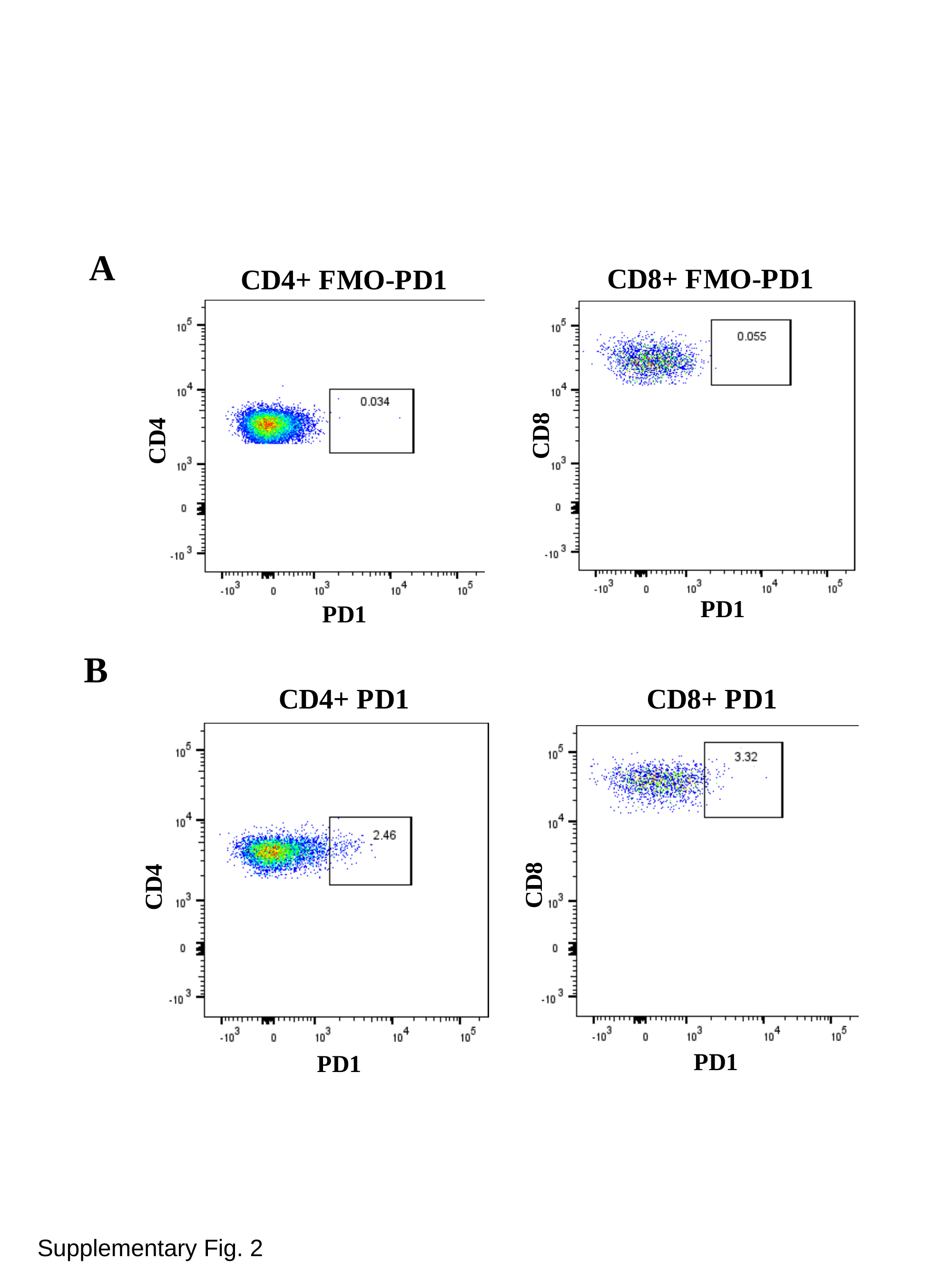

Supplementary Fig. 2

Supplement: Supplementary file 1 — Supplementary file1 Fig. 1 Flow chart of the study population. A total of 133 PBMC samples was obtained from 115 NAFLD and AIH patients and 18 controls. Liver biopsy was performed on the 115 patients at the University of Tokyo; not on controls. Among the patients, 88 were diagnosed with NAFLD, 27 with AIH (acute:chronic = 12:15). Regarding NAFLD, 70 patients were diagnosed as NASH, 18 as NAFL. The 70 NASH patients were divided into three groups: ANA negative, 36; ANA positive, 20; and AIH-overlap, 14., FCM plots and gating strategies used to identify PD1+ T cells: (A) FMO for CD4+ PD1+ (left) and CD8+ PD1+ T cells (right); (B) gated on CD4+ PD1+ (left) and CD8+ PD1+ T cells (right). Data are from one healthy control. (PPTX 115 KB) [file 535_2023_2038_MOESM1_ESM.pptx]
