# Supplementary material for: Noninvasive approach to indicate risk factors of nonalcoholic steatohepatitis overlapping autoimmune hepatitis based on peripheral lymphocyte pattern
Source: J Gastroenterol. 2023 Sep 14;58(12):1237–51. doi: 10.1007/s00535-023-02038-y (PMC10657798; doi:10.1007/s00535-023-02038-y)
Supplement: Supplementary file 2 — Supplementary file2 (DOCX 28 KB) [file 535_2023_2038_MOESM2_ESM.docx]

**Abbreviations:**

PBMC peripheral blood mononuclear cells

FBS Fetal Bovine Serum

FACS Fluorescence-activated cell sorting

DPBS Dulbecco’s phosphate-buffered saline

NK natural killer

Th helper T cell]

Treg regulatory T cells

PD programmed cell death

CTLA cytotoxic T-lymphocyte antigen

FMO fluorescence minus one

SSC side scatter area

FSC forward scatter area

CXCR C-X-C chemokine receptor

CCR C-C chemokine receptor

**Supplementary Methods**

*Cell culture and surface staining*

Peripheral blood mononuclear cells (PBMCs) were suspended in 10 mL RPMI 1640 medium (Merck, Darmstadt, Germany) supplemented with penicillin (100 U/mL), streptomycin (100 μg/mL), L-glutamine, and 10% fetal bovine serum (FBS) after two washes (1500 g, 5 min, room temperature). They were adjusted to 5.0 × 10^5^/mL and cultured in 10 mL RPMI 1640 medium at 37°C with 5% CO_2_ for 24 h. Next, they were suspended in cold fluorescence-activated cell sorting (FACS) buffer supplemented with Dulbecco’s phosphate-buffered saline (DPBS) (Merck), 2% FBS, and 0.02% sodium azide. Sodium azide was used to prevent the modulation and internalization of surface antigens, which can produce a loss of fluorescence intensity. PBMCs were stained for 30 min at 4°C in the dark with the 1:1000 LIVE/DEAD Fixable Aqua Dead Cell Stain Kit (Thermo Fisher Scientific, MA) to determine cell viability prior to formaldehyde fixation and permeabilization for intracellular antibody staining and elimination of biohazardous materials.

After one wash, PBMCs were resuspended in cold FACS buffer, and monoclonal antibodies were added for surface staining of human peripheral lymphocytes based on two 11-color antibody cocktails. Cocktail I was used to identify lymphocyte populations (T cells, B cells, natural killer [NK] cells, and NKT cells), and Cocktail II was used to identify T cell subsets (helper T cell [Th] 1, Th2, Th17, regulatory T cells [Treg], and programmed cell death [PD] 1^+^ or cytotoxic T-lymphocyte antigen [CTLA] 4^+^ cells among CD4^+^ and CD8^+^ T cells) (Supplementary Table 1). The fluorochrome-labeled antibodies were selected based on the optimal signal strength and minimal spectral overlap; the concentration of each antibody was determined by titration. PBMCs were incubated for 30 min on ice in the dark. Unstained, isotype, and fluorescence-minus-one (FMO) controls were included to set gate limits and identify antibody nonspecific binding to the cell surface. After two washes, PBMCs were resuspended in cold FACS buffer, 4% paraformaldehyde was added for fixation and prevention of deterioration, and then the cells were incubated for 15 min at room temperature in the dark.

*FCM gating strategies and flow cytometric analysis*

FCM data were exported and analyzed using FlowJo version 10.8.1 (Treestar). To determine positive/negative cut-offs for the gating strategy, we included FMO controls [1]; PBMC samples were stained with all but one of the reagents, enabling identification of cells with greater-than-background fluorescence [1,2]. PBMC samples were examined by side scatter area (SSC-A) versus forward scatter area (FSC-A), and forward scatter height (FSC-H) versus FSC-A and side scatter height (SSC-H) versus SSC-A were applied to select single cells and eliminate debris and clumped cells [3]. Single cells were sub-gated using the LIVE/DEAD Fixable Aqua Dead Cell Stain Kit (Thermo Fisher Scientific) to exclude dead cells; subsequently, live cells were discriminated by the expression of CD45, a leukocyte surface marker [4]. CD45 receptors on lymphocytes are stable over time, and the light-scattering discriminator can eliminate unwanted contaminants from the analysis; therefore, CD45-based gating can be used to detect lymphocytes.

In this study, FCM plots and gating strategies were used to identify target lymphocytes (Fig. 1). First, peripheral lymphocytes were examined; aggregated cells were excluded, followed by the selection of single and viable cells after amine staining of non-viable cells. Next, CD45^+^ leukocytes were selected, followed by CD3^+^ T cells (CD3/SSC-A gating), CD4^+^ CD8^−^ and CD4^−^ CD8^+^ T cells (CD4/CD8 gating), CD3^−^ CD19^+^ B cells (CD3/CD19 gating), CD3^−^ CD56^+^ NK cells (CD3/CD56 gating), and CD3^+^ CD56^+^ NKT cells (CD3/CD56 gating) (Fig. 1A). Regarding CD4/CD8 gating, CD4^+^ and CD8^+^ cells are respectively mixed with dim and bright cells, which might be caused by the compensation issue; therefore, bright cells were selected for analysis as much as possible. The fluorochrome-labeled antibodies in Cocktail I (Supplementary Table 1) were used for these gating strategies.

CD3^+^ T cells were selected, followed by CD4^+^ T cells (CD4/CD8 gating). Among the CD4^+^ T cell populations, CD25^−^ C-X-C chemokine receptor (CXCR) 3^+^ T cells were selected by CD25/CXCR3 gating, followed by selection of C-C chemokine receptor (CCR) 4^−^ CCR6^−^ Th1 (CCR4/CCR6 gating), CD25^−^ CXCR3^−^ T cells (CD25/CXCR3 gating), and CCR4^−^ CCR6^+^ Th2 and CCR4^+^ CCR6^+^ Th17 cells (CCR4/CCR6 gating) (Fig. 1B). CD25^+^ CD127^−^ Tregs were selected by CD25/CD127 gating. Furthermore, as surface markers of immune tolerance on T cells, PD1 and CTLA4 were examined. Among the CD4^+^ and CD8^+^ T cell populations, respectively, PD1^+^ CTLA4^−^ and PD1^−^ CTLA4^+^ cells were selected by PD1/CTLA4 gating (Fig. 1C); especially, the frequency of PD1^+^ cells, an important indicator in this study, tended to be considerably low and was calculated carefully to maintain fairness and continuity of FCM gating among all PBMC samples with FMO as shown in Supplementary Fig. 2. The fluorochrome-labeled antibodies in Cocktail Ⅱ (Supplementary Table 1) were used for these gating strategies.

CD45 can be used to determine the ratios of T cells, B cells, NK cells, NKT cells, and CD4^+^ T cells to assess Th subsets. PD1- and CTLA4-positive T lymphocyte frequencies were calculated by CD4- and CD8-based gating. In this study, target lymphocytes in 133 PBMC samples were selected using the gating strategies in Fig. 1, and their frequencies were calculated.

*Statistical analysis*

Data were analyzed using JMP 16 software (SAS Institute Japan, Tokyo, Japan). A value of *p* < 0.05 was considered indicative of statistical significance. Nonparametric data were analyzed using the Wilcoxon Kruskal–Wallis test. Data are medians and interquartile ranges (lower to upper quartile) unless stated otherwise. Univariate logistic regression was used to identify predictors of NASH and autoimmunity-related NASH. We examined variables that differed significantly between NASH and NAFL; between ANA-negative and other types of NASH; and between ANA-positive and AIH-overlap NASH. Multivariable logistic regression was performed to test variables with *p* < 0.02 in univariate analyses. We selected models using the Akaike Information Criterion.

**Supplementary References**

1. Perfetto SP, Chattopadhyay PK, Roederer M. Seventeen-colour flow cytometry: Unravelling the immune system. Nat Rev Immunol. 2004; 4: 648–55.

2. Maecker HT, Trotter J. Flow cytometry controls, instrument setup, and the determination of positivity. Cytometry A. 2006; 69: 1037–42.

3. Mensurado S, Rei M, Lança T, et al. Tumor-associated neutrophils suppress pro-tumoral IL-17+ γδ T cells through induction of oxidative stress. PLoS Biol. 2018; 16: e2004990.

4. Janossy G, Jani IV, Bradley NJ, et al. Affordable CD4+ T-cell counting by flow cytometry: CD45 gating for volumetric analysis. Clin Diagn Lab Immunol. 2002; 9: 1085–94.
