# Supplementary material for: Noninvasive approach to indicate risk factors of nonalcoholic steatohepatitis overlapping autoimmune hepatitis based on peripheral lymphocyte pattern
Source: J Gastroenterol. 2023 Sep 14;58(12):1237–51. doi: 10.1007/s00535-023-02038-y (PMC10657798; doi:10.1007/s00535-023-02038-y)
Supplement: Supplementary file 3 — Supplementary file3 (DOCX 48 KB) [file 535_2023_2038_MOESM3_ESM.docx]

**Supplementary Table 1** Antibodies used in two cocktails, specified by antibody, fluorochrome, clone, isotype, and source

The correlations between peripheral lymphocyte frequencies and several parameters: three components of NAS, fibrosis, and the levels of AST, ALT, and IgG, were examined.

Associations with p < 0.05 are bold.

**Supplementary Table 2** Changes in peripheral lymphocyte frequencies of 70 NASH patients according to the degree of NAS, fibrosis, and the levels of AST, ALT, and IgG

Values are presented as median (P25-P75)

**Supplementary Table 3** Laboratory parameters and peripheral lymphocyte frequencies before and after steroid therapy for 9 AIH-overlap NASH patients

*p<0.05 AIH-overlap NASH compared with acute AIH, **p<0.001 AIH-overlap NASH compared with acute AIH

#p<0.05 AIH-overlap NASH compared with chronic AIH, ##p<0.001 AIH-overlap NASH compared with chronic AIH

Values are presented as N, N (%), or median (P25-P75)

**Supplementary Table 4** Clinical characteristics and peripheral lymphocyte frequencies between 27 AIH and 14 AIH overlapping NASH patients

*p<0.05 compared with controls, **p<0.001 compared with controls

Values are presented as median (P25-P75)

**Supplementary Table 5** Peripheral lymphocyte frequencies between 27 AIH patients and 18 healthy controls
